# Dynamic Validation of Calibration Accuracy and Structural Robustness of a Multi-Sensor Mobile Robot

**DOI:** 10.3390/s24123896

**Published:** 2024-06-16

**Authors:** Yang Liu, Ximin Cui, Shenghong Fan, Qiang Wang, Yuhan Liu, Yanbiao Sun, Guo Wang

**Affiliations:** 1 School of Geosciences and Surveying Engineering, China University of Mining and Technology (Beijing), Beijing 100083, China; bqt1800204050@student.cumtb.edu.cn (Y.L.); cxm@cumtb.edu.cn (X.C.); 2 Beijing Prodetec Technology Co., Ltd., Beijing 100083, China; fanshenghong@prodetec.cn; 3 Faculty of Geography, Tianjin Normal University, Tianjin 300387, China; 2210080046@stu.tjnu.edu.cn; 4 State Key Laboratory of Precision Measuring Technology and Instruments, Tianjin University, Tianjin 300072, China; yanbiao.sun@tju.edu.cn; 5 Institute of Civil Engineering, Henan University of Engineering, Zhengzhou 451191, China; wg@haue.edu.cn

**Keywords:** multi-sensor, mobile robot, calibration accuracy, structural robustness, dynamic validation

## Abstract

For mobile robots, the high-precision integrated calibration and structural robustness of multi-sensor systems are important prerequisites for ensuring healthy operations in the later stage. Currently, there is no well-established validation method for the calibration accuracy and structural robustness of multi-sensor systems, especially for dynamic traveling situations. This paper presents a novel validation method for the calibration accuracy and structural robustness of a multi-sensor mobile robot. The method employs a ground–object–air cooperation mechanism, termed the “ground surface simulation field (GSSF)—mobile robot -photoelectric transmitter station (PTS)”. Firstly, a static high-precision GSSF is established with the true north datum as a unified reference. Secondly, a rotatable synchronous tracking system (PTS) is assembled to conduct real-time pose measurements for a mobile vehicle. The relationship between each sensor and the vehicle body is utilized to measure the dynamic pose of each sensor. Finally, the calibration accuracy and structural robustness of the sensors are dynamically evaluated. In this context, epipolar line alignment is employed to assess the accuracy of the evaluation of relative orientation calibration of binocular cameras. Point cloud projection and superposition are utilized to realize the evaluation of absolute calibration accuracy and structural robustness of individual sensors, including the navigation camera (Navcam), hazard avoidance camera (Hazcam), multispectral camera, time-of-flight depth camera (TOF), and light detection and ranging (LiDAR), with respect to the vehicle body. The experimental results demonstrate that the proposed method offers a reliable means of dynamic validation for the testing phase of a mobile robot.

## 1. Introduction

As the field of autonomous driving continues to evolve, mobile robots with high mobility, such as ground mobile robotic vehicles and lunar rovers, play a pivotal role in mission execution [1,2]. These mobile robots are equipped with a variety of sensors, including navigation cameras (Navcams), hazard avoidance cameras (Hazcams), light detection and ranging (LiDAR), time-of-flight depth cameras (TOF), inertial measurement units (IMU), and others, which enable them to gather diverse information about the surroundings. This information plays a critical role in a number of tasks, including navigation and positioning, environmental perception, mapping and modeling, and scientific experiments [3,4,5]. For mobile robots, such as ground mobile robots, the bumps or vibrations generated during the movement process and the limitations of their own structure can easily cause changes in the mechanical structure of the aforementioned sensor system. This can result in the deformation of the robot chassis or sensor mounts, which in turn affects the acquisition of environmental information. Consequently, in the context of a multi-sensor system for ground mobile robots, ensuring the precise calibration of various sensors and the robustness of the sensor system’s structure represents a pivotal step in guaranteeing the consistency of data fusion and the overall performance of the system.

A substantial corpus of research has been conducted on the topic of sensor calibration. These include camera-to-camera calibration [6], camera-to-LiDAR calibration [7], camera-to-IMU calibration [8], and calibration between different sensors and vehicle bodies [9]. Calibration serves to elucidate the relative relationships between sensors, and the absolute relationships between certain sensors and the vehicle body are clarified, which is of paramount importance for subsequent applications of multi-sensor fusion. It is inevitable that sensor calibration will necessitate the use of calibration models or methods. To ensure the accuracy and reliability of calibration, many researchers conduct extensive experiments on the selection of calibration models or methods, such as repeated experiments, noise addition, and so forth, to evaluate the robustness of the calibration method. The aforementioned accuracy validations pertain to the calibration methods themselves, originating from the methodology. In addition to evaluating the accuracy of intrinsic and extrinsic calibration parameters, it is also essential to assess the robustness of the multi-sensor mechanical structure, given the intrinsic and extrinsic calibration parameters.

The majority of the existing literature concentrates on the validation of the accuracy and robustness of intrinsic parameters for individual sensors, as well as the calibration accuracy of relative orientation parameters (ROPs) between multiple sensors. Nevertheless, there is a paucity of research reports on the pertinent aspects of the validation methods for the exterior orientation parameters (EOPs) and structural robustness of the sensors with respect to the vehicle body in the traveling state. In more precise terms, there has been no coverage of comprehensive accuracy validation for various sensors on mobile robots, particularly dynamic validation methods for the overall calibration accuracy and structural robustness of sensor systems for mobile robots. Consequently, the objective of this study is to propose a comprehensive and efficacious dynamic validation method for the calibration accuracy and structural robustness of a multi-sensor mobile robotic.

**Figure 1:**
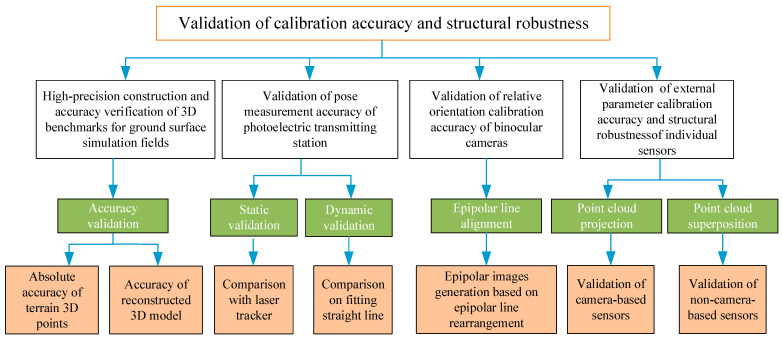
Validation architecture for calibration accuracy and structural robustness of a multi-sensor mobile robot.

The system architecture design for the dynamic validation of the calibration accuracy and structural robustness of a multi-sensor mobile robotic vehicle is depicted in Figure 1. Dynamic validation involves two important aspects: the first is to verify the accuracy of pose tracking and measuring of the vehicle body by the photoelectric transmitting station (PTS), and the second is to verify the absolute calibration accuracy and structural robustness of each sensor. Verification of the absolute calibration accuracy and structural robustness of each sensor is not possible without the support of a high-accuracy three-dimensional (3D) reference field. Consequently, the subsequent research presented in this paper is divided into four parts. The first part involves the high-precision construction and accuracy validation of a 3D digital reference from a ground surface simulating field (GSSF). The second part concerns the pose tracking measurement of the vehicle body using high-precision PTS, with the objective of validating the accuracy of this method. The third part is dedicated to the validation of the calibration accuracy of ROPs of the stereo camera. The fourth and final part of this research is the dynamic validation of the calibration accuracy of EOPs and the structural robustness of each individual sensor.

## 2. Related Works

The evaluation or validation of sensor calibration accuracy can be broadly categorized into two types: One type focuses on the evaluation or validation of calibration accuracy from the aspect of the calibration method itself. The other type focuses on the evaluation or validation of calibration accuracy from the aspect of the calibration results.

### 2.1. Accuracy Validation Based on the Calibration Method

When evaluating or verifying the calibration accuracy of a sensor’s internal and external parameters from the perspective of the calibration method itself, two main methods are commonly used to evaluate the accuracy. The first method is to observe the magnitude of the effect of image noise variations on the calibration results. Batista et al. [10] verified the robustness of the proposed iteration-based calibration method for a perspective camera under different noise levels. While evaluating the magnitude of the effect of noise variations on the calibration results allows for the examination of the robustness of the calibration results, it may not be able to fully capture all the sources of error, particularly given that the variations in noise in a real environment are often influenced by multiple factors. A second method is to compare with other mature calibration methods. Du et al. [11] compared the accuracy of multi-camera calibration based on the Seagull algorithm with the classical Zhang’s calibration method by computing the mean and standard deviation of projection errors. Xu et al. [12] proposed a novel external calibration procedure based on six degrees of freedom (DoF) and, by comparing it with the classical Kalibr calibration method, achieved an absolute attitude error of less than 0.025°. While comparisons with established calibration methods can provide a reference point, such comparisons may be influenced by differences or limitations among different methods.

### 2.2. Accuracy Validation Based on the Calibration Results

When evaluating or verifying the calibration accuracy of the sensor’s interior parameters and EOPs from the perspective of the calibration results, it can also be categorized into two main methods: one method is to compare it with the known high-precision true value, and another method is an indirect verification based on the post-processing results by the geometrical model.

Regarding the method that compares with the known high-precision true value, Kim et al. [13] proposed a framework for vehicle detection based on deep learning. The accuracy of trajectory calibration is verified by comparing manually marked vehicle positions with calibrated vehicle positions. In the context of calibrating a multi-sensor vision measurement system based on structured light, Zhang et al. [14] employed images and 3D coordinates captured by multiple sensors to validate the accuracy of global calibration. While comparison with known ground truth values is a reliable method, it may not be applicable in all scenarios, and it can be influenced by calibration methods and equipment errors. Regarding the method that indirectly verifies based on the post-processing results of the geometrical model, it employs mathematical models and algorithms that are based on the geometrical shapes, features, and motion patterns of objects. This approach not only comprehensively considers error sources and system characteristics but also provides accurate calibration accuracy assessment in the absence of real values as a reference. The indirect verification based on post-processing results with a geometric model refers to the use of preset projection models or theoretical geometric relationships. These include the projected point positions after projection and the homonymous point positions after epipolar correction, which should have a specific geometric relationship. This is used to assess the precision and accuracy of the calibration parameters by analyzing the differences between the actual projected positions and the theoretically expected positions, as well as the differences in the longitudinal coordinates between the homonymous points, respectively. For example, Semeniuta [15] evaluated the quality of calibrated camera parameters by assessing the performance of stereo triangulation. Projection error refers to the discrepancy between the actual 2D pixel coordinates and the estimated 2D pixel points. This error is often expressed using metrics such as the root mean square root (RMSE) and the average projection error. In a study by Liu et al. [16], the accuracy of a multi-camera stereo calibration method based on circular calibration boards was evaluated by calculating projection errors. Zhang [17] analyzed the calibration accuracy of the camera with LiDAR and IMU by calculating the projection error and orientation error, which were composed of the theoretical and actual values.

In general, the indirect validation method based on the post-processing results with the geometric model is more realistic and has been applied to the verification of calibration accuracy of multi-sensor systems such as unmanned aerial vehicles (UAVs) and unmanned ships. In a study by Pentek et al. [18], the relative relationships of LiDAR, global navigation satellite system (GNSS)/inertial navigation system (INS), and camera were calibrated and validated on a drone platform. Additionally, studies have been conducted to establish a unified spatial coordinate system among multiple sensors for calibration accuracy validation. For instance, Jing et al. [19] unified above-water and underwater sensors (3D laser scanners, multibeam sounders, GNSS, and IMU) into the same coordinate system for calibration accuracy validation. This approach is more comprehensive, providing more comprehensive and accurate calibration results, thereby ensuring better overall system performance. However, the aforementioned method is only suitable for the dynamic verification of the relative relationship between these sensors. It is not possible to carry out an absolute dynamic verification of the relationship between the individual sensors with respect to the coordinate system of the carrier body. Consequently, these methodologies remain unsuitable for the dynamic absolute validation of sensors for mobile robots.

## 3. Materials

**Figure 2:**
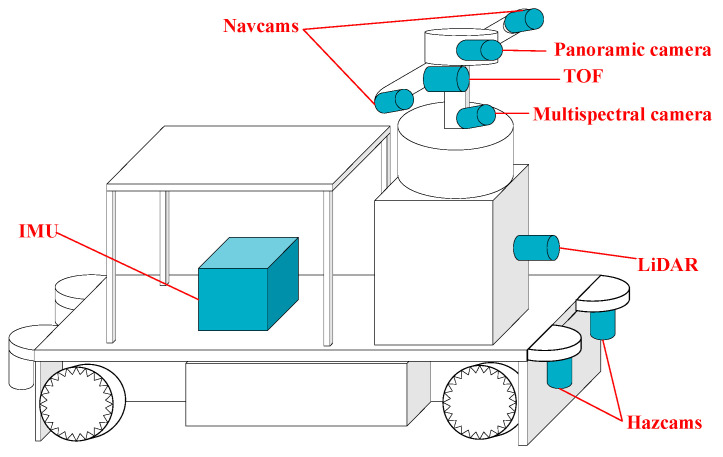
Multi-sensor system of a mobile vehicle.

The mobile robot is a mobile robotic vehicle, and the sensor layout is highly compact. The multi-sensor mobile robot is equipped with a variety of mapping and remote sensing sensors, including a pair of Navcams, a pair of Hazcams, a multispectral camera, a LiDAR, a TOF, an IMU, and a panoramic camera (Figure 2).

**Figure 3:**
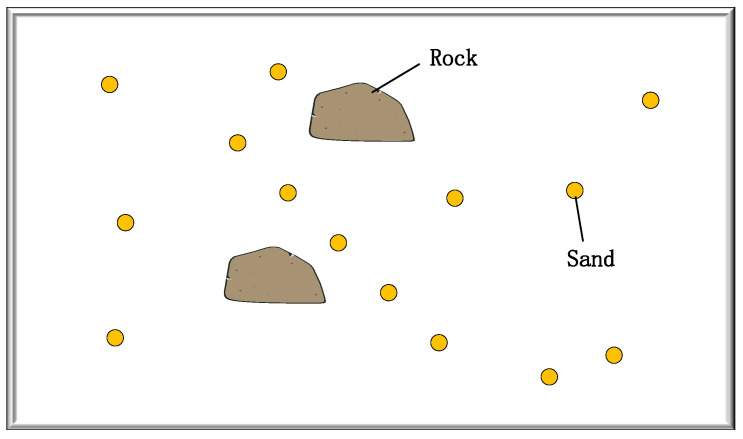
Diagram of the ground surface simulated field.

An experimental GSSF acquired by ground laser scanner is used to explore the effectiveness and feasibility of dynamic validation techniques for the calibration accuracy and structural robustness of multi-sensor mobile robots. Figure 3 shows the diagram of the experimental GSSF, which is filled with sand and rocks.

The sensors utilized for validation include Navcams, Hazcams, multispectral cameras, TOF, and LiDAR, which are operational in this experiment. Navcams, Hazcams, and multispectral cameras are classified as camera-based sensors, as their primary mode of presentation is image data. TOF and LiDAR are classified as non-camera-based sensors due to the predominant presentation of 3D point cloud data.

**Figure 4:**
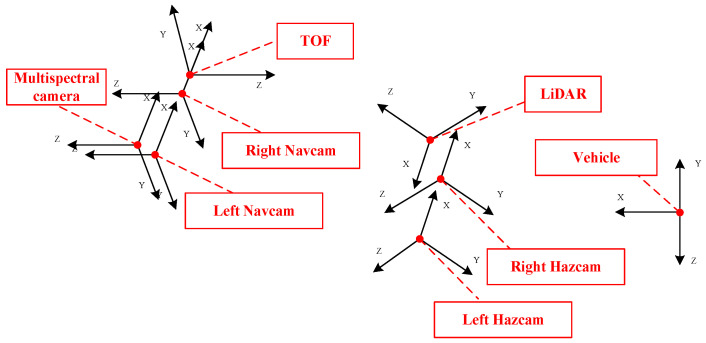
Three-dimensional display of each sensor coordinate system based on vehicle body coordinate system.

Following preliminary work, calibration for each sensor on the multi-sensor mobile robot has been completed. This involved obtaining the EOPs of each sensor based on the vehicle coordinate system as a reference. Figure 4 shows the 3D representation of the relationships between various coordinate systems.

## 4. Methods

**Figure 5:**
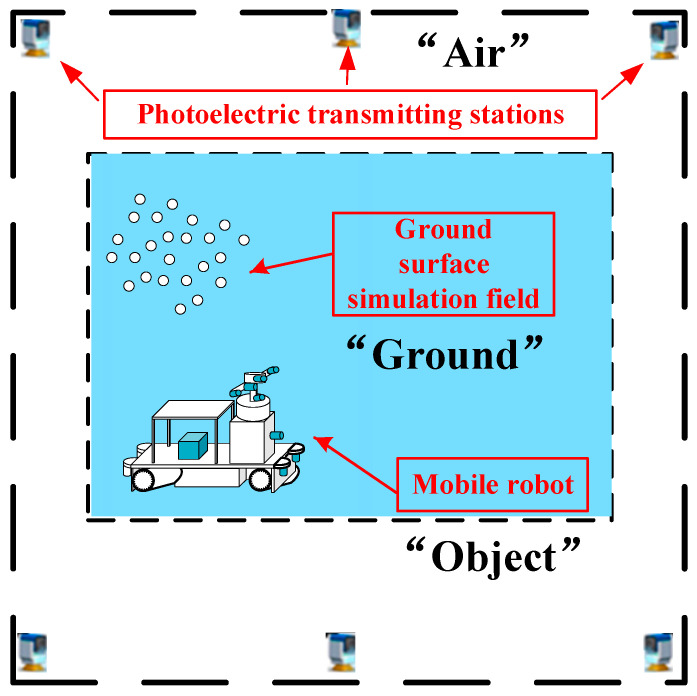
The ground–object–air cooperation mechanism of “GSSF-Robot-PTS”.

The core of the proposed dynamic validation method for calibration accuracy and structural robustness is based on the ground–object–air cooperation mechanism of “GSSF-Mobile robot-PTS” (Figure 5). The term “Object” represents the mobile robot, the term “Air” represents the photoelectric transmitting station on the roof, and the term “Ground” represents the stationary ground surface simulation field. The mobile robot and the PTS are linked in a dynamic manner, while the GSSF remains static. In the event that the mobile robot is not situated within the field of view of the PTS, the latter will be prompted to move in order to track the former. The GSSF is established in the true north reference coordinate system, and the measurement coordinate system of the PTS is transformed into the true north reference coordinate system in real time. This transformation ensures that the mobile robot observed by the PTS is also transformed to the true north coordinate system in real time. Given the relative relationship between the sensors mounted on the mobile robot and the robot body is known, the sensor coordinate system can be correlated with the true north coordinate system and the GSSF. Subsequently, the data from the GSSF can then be employed to validate the calibration accuracy and structural robustness of each individual sensor in a dynamic manner. In other words, by back-projecting the point cloud of the GSSF onto the camera plane, the accuracy of the calibration relationship between the camera sensor and the vehicle body, as well as the structural robustness between the two, can be tested. Similarly, by superimposing the point cloud of the GSSF with the LiDAR point cloud or the TOF point cloud, we can test the accuracy of the calibration relationship between the non-camera sensor and the vehicle body, and the structural robustness between the two can be evaluated.

In the realization phase, a laser scanner was employed to obtain a multi-station point cloud, which was subsequently stitched together to form a complete GSSF. The accuracy of this process was then evaluated. The measurement accuracy of the PTS was verified in two ways. First, it was compared with the vehicle body position solved by the laser tracker. Second, the linear trajectory measurement of the slider was used to verify the dynamic accuracy of the PTS. The ROPs calibrated by the binocular camera were verified based on the epipolar line realignment. Finally, the EOPs calibration accuracy and structural robustness of the navigation, hazard avoidance, and multispectral camera-based sensors, as well as the TOF and LiDAR non-camera-based sensors, were verified by re-projecting the 3D point cloud onto the image plane and point cloud superposition, respectively.

### 4.1. The Accuracy Validation Method for the GSSF

#### 4.1.1. Laser Point Cloud Acquisition for GSSF

Three-dimensional laser scanning is an efficient and high-precision non-contact active measurement technique capable of rapidly acquiring high-precision, high-density 3D point clouds of object surfaces. The process of indoor mapping based on ground-based laser scanners typically involves two steps of point cloud registration. The first step is a coarse registration based on pre-arranged regular markers or reference spheres [20,21]. The second step is to refine the coarse results by feature-based registration.

**Figure 6:**
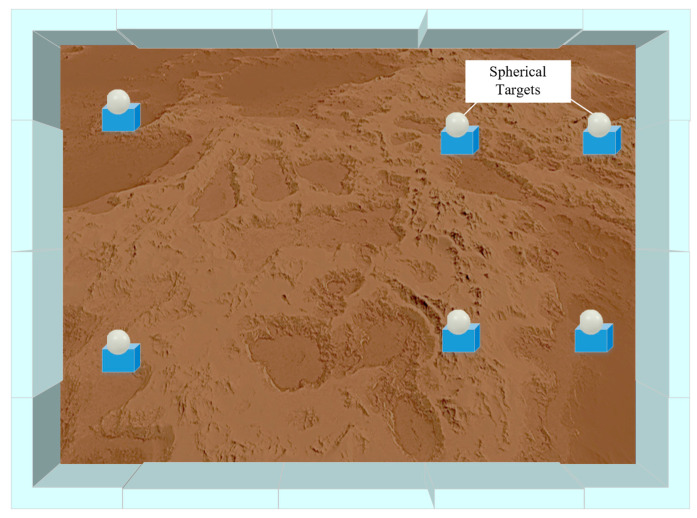
Arrangement of six spherical targets.

Six spherical targets were uniformly placed in the simulation field at the obvious elevated places, and the boxes represent the positions of the six spherical targets. The on-site arrangement of the spherical targets is shown in Figure 6.

**Figure 7:**
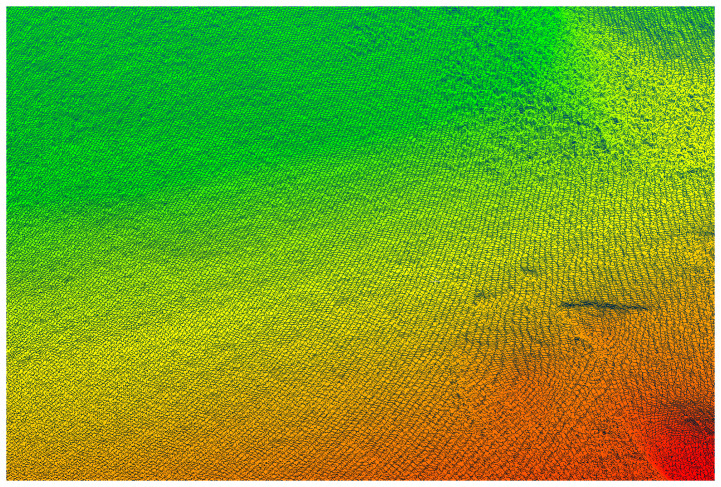
Laser scanning point cloud of the simulation field.

The complete point cloud of the whole GSSF can be obtained by filtering and registering the measured point cloud, as illustrated in Figure 7. The point cloud is converted by employing the relative relationship between the laser scanner coordinate system and the true north coordinate system, thereby enabling the acquisition of the GSSF under the true north datum.

#### 4.1.2. Accuracy Evaluation of the Reconstructed 3D GSSF

The accuracy of the 3D simulation field is evaluated in terms of both the absolute accuracy of the 3D terrain points and the accuracy of the reconstruction model.

(a) Absolute accuracy of 3D terrain points

Spherical targets are distributed uniformly throughout the terrain field in order to represent the 3D points of the terrain. The accuracy of the coordinates of the terrain 3D points is to be evaluated in comparison with the theodolite system. The scanner is capable of automatically recognizing spherical targets and calculating the coordinates of the center of the sphere. In contrast, the theodolite system employs a different approach, whereby the center positions are fitted by matching the edges of the spherical targets. Consequently, the centers of the spherical targets can be employed as common points for both systems.

**Figure 8:**
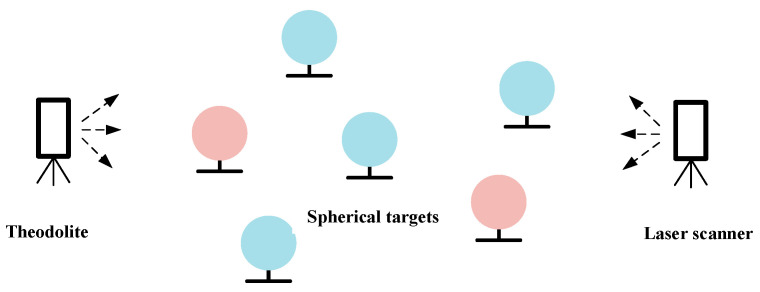
The deployment of spherical targets for accuracy test of terrain 3D points.

The test method is as follows: Establish a theodolite coordinate system and place four spherical targets in the GSSF as common points for the conversion between the true north coordinate system of the laser scanning point cloud and the theodolite coordinate system. Subsequently, two additional spherical targets should be placed in the simulation field multiple times (re-scan the simulation field at each time) as randomly sampled points. The on-site layout is schematically shown in Figure 8. The coordinates of the spherical targets in the two systems were measured, and the 3D absolute accuracy of the terrain points was calculated by comparing the deviations.

x, y, and z represent the coordinates of the target center measured by the theodolite system, and $x^{\prime },y^{\prime }$, and $z^{\prime }$ represent the coordinates of the target center measured by the laser scanner system. The absolute accuracy $E_{p}$ of the terrain 3D points is calculated according to Equation (1).

(1) $$E_{p}=\sqrt{{\left(x-x^{\prime }\right)}^{2}+{\left(y-y^{\prime }\right)}^{2}+{\left(z-z^{\prime }\right)}^{2}}$$

(b) Accuracy of the reconstructed model

**Figure 9:**
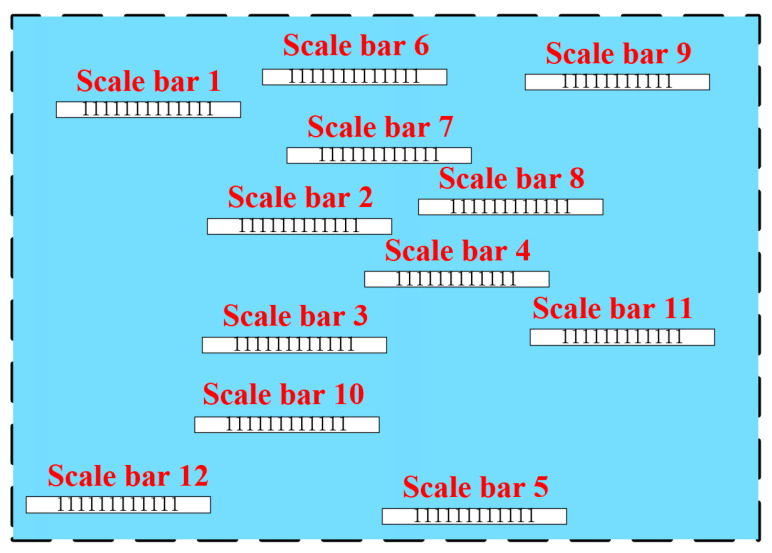
The deployment of scale bars for accuracy test of statically reconstructed terrain.

The test method is as follows: The standard scale bar is uniformly positioned within the GSSF, and the terrain model (in mesh format) is reconstructed. The length of each scale bar was measured and compared with their real calibrated length to calculate the accuracy of the statically reconstructed terrain, as illustrated in Figure 9.

By calculating the RMSE between the nominal length and the actual reconstructed length of the standard scale bars, the accuracy of the reconstructed model is assessed.

(2) $$E_{rms}=\sqrt{\frac{1}{n}\sum_{i=1}^{n}{\left(L^{\prime }-L\right)}^{2}}$$

In Equation (2), *n* represents the number of scale bars. L represents the nominal length of the standard scale bar, $L^{\prime }$ is the reconstructed measured length, and $E_{rms}$ is the error.

### 4.2. The Accuracy Validation Method for Pose Measurement of the PTS

**Figure 10:**
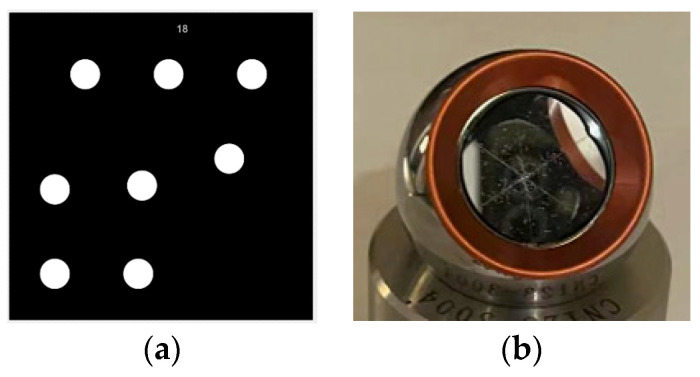
Target attached to the robot. (**a**) Coded targets; (**b**) reflective spherical prism target.

The methods for pose measurement are primarily divided into two categories: contact measurement, which involves the use of a robotic arm with a probe [22], and non-contact measurement, which employs inertial navigation systems, photogrammetry, and laser tracking measurement [23]. Photogrammetry and laser tracking measurement are capable of tracking spatial targets in real time and measuring the spatial 3D coordinates of the targets. In this paper, the photogrammetric method based on the PTS is employed to track and measure the vehicle body’s pose in real time, with a laser tracker serving as the benchmark for high-precision pose measurement. The PTS is equipped with a ring light source and a laser rangefinder, whose key device is a high-resolution camera with a resolution of 5120 × 5120. The photogrammetric method relies on the attachment of coded targets to the vehicle body (Figure 10a), while the laser tracker method relies on mounting a base and prism to the vehicle body (Figure 10b). The coded target is a special retro-reflective marker that offers several advantages, including high recognition robustness, automatic numbering, and high positioning accuracy [24]. Consequently, the coded target is an optimal choice for attachment to the vehicle body for tracking and localization purposes.

#### 4.2.1. Real-Time Pose Tracking Measurement of Vehicle Body Based on Step-by-Step Photogrammetric Approach

A fundamental prerequisite for utilizing the PTS for real-time pose tracking measurement is the acquisition of the 3D coordinates of the coded targets on the vehicle body in the true north coordinate system. The theoretical framework is based on the step-by-step measurement method in photogrammetry, which is known as spatial resection-forward intersection.

(3) $$\begin{array}{c} x+\Delta x^{\prime }=-f\frac{a_{1}\left(X-X_{S}\right)+b_{1}\left(Y-Y_{S}\right)+c_{1}\left(Z-Z_{S}\right)}{a_{3}\left(X-X_{S}\right)+b_{3}\left(Y-Y_{S}\right)+c_{3}\left(Z-Z_{S}\right)}\\ y+\Delta y^{\prime }=-f\frac{a_{2}\left(X-X_{S}\right)+b_{2}\left(Y-Y_{S}\right)+c_{2}\left(Z-Z_{S}\right)}{a_{3}\left(X-X_{S}\right)+b_{3}\left(Y-Y_{S}\right)+c_{3}\left(Z-Z_{S}\right)} \end{array}\}$$

Equation (3) represents a non-linear collinearity equation. In order to perform least squares adjustment processing, the equation must be linearized. The correction equation for the linearized image coordinates is as follows:

(4) $$V=A_{1}X_{1}+A_{2}X_{2}-L$$

where

$$V=\left[\begin{array}{c} v_{x}\\ v_{y} \end{array}\right],\;A_{1}=\left[\begin{array}{cccccc} c_{11} & c_{12} & c_{13} & c_{14} & c_{15} & c_{16}\\ c_{21} & c_{22} & c_{23} & c_{24} & c_{25} & c_{26} \end{array}\right],\;L={\left[\begin{array}{c} x-\bar{x}\\ y-\bar{y} \end{array}\right]}^{T},\;A_{2}=\left[\begin{array}{ccc} -c_{11} & -c_{12} & -c_{13}\\ -c_{21} & -c_{22} & -c_{23} \end{array}\right],\;X_{2}={\left[\begin{array}{ccc} \Delta X & \Delta Y & \Delta Z \end{array}\right]}^{T}\;\;\mathrm{and}\;X_{1}={\left[\begin{array}{cccccc} \Delta X_{S} & \Delta Y_{S} & \Delta Z_{S} & \Delta \omega & \Delta \varphi & \Delta \kappa \end{array}\right]}^{T}.$$

Each image covers a certain number of coded targets. Based on the collinearity equation, the EOPs for each image can be solved using a single-image space resection. The coordinates of the control point can be regarded as true values (i.e., $X_{2}$ = 0), and only the EOPs $X_{1}$ need to be solved. Equation (4) becomes

(5) $$V=A_{1}X_{1}-L$$

For each control point, two equations can be formulated according to Equation (5). The values of the six EOPs of the image can be determined by using more than three control points.

Through the aforementioned space resection, the pose of the PTS in the true north coordinate system can be calculated. Subsequently, the recognition of the vehicle’s coded targets can be performed, and their coordinates in the true north coordinate system can be calculated through forward intersection. Specifically, in Equation (4), after setting $X_{1}=0$, then

(6) $$V=A_{2}X_{2}-L$$

For each corresponding image point of the object point, two equations can be formulated according to Equation (6). Consequently, when an object point is imaged on two or more photos, its coordinates can be determined through the least squares adjustment.

**Figure 11:**
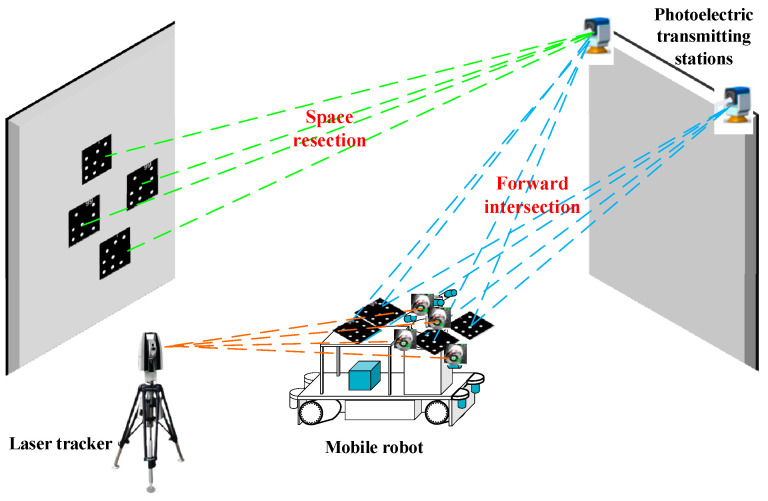
Schematic diagram of pose measurement of mobile robot.

The process of implementing the pose measurement of the vehicle by the PTS is as follows:

(1) A specific number of coded targets are placed around the GSSF and on the vehicle body. The coordinates of the coded targets situated around the GSSF are measured in advance and transformed into the true north coordinate system. The coded targets on the vehicle are tracked and measured by the PTS to obtain the pose of the vehicle.
(2) The vehicle moves in the GSSF, and the PTS takes continuous photos of the GSSF and the vehicle. The photogrammetric measurement method is employed in a step-by-step manner by the PTS to obtain the vehicle’s pose by the PTS, as illustrated in Figure 11. Specifically, the coded targets around the GSSF serve as control points for the purpose of space resection, thereby enabling the PTS to obtain the EOPs in the true north coordinate system. Subsequently, multiple PTSs can utilize multi-image space-forward intersections to determine the 3D coordinates of each coded target on the vehicle in the true north coordinate system. The vehicle body coordinate system can be recovered by more than three coded targets, thus enabling the pose of the vehicle in the true north coordinate system to be determined.

**Figure 12:**
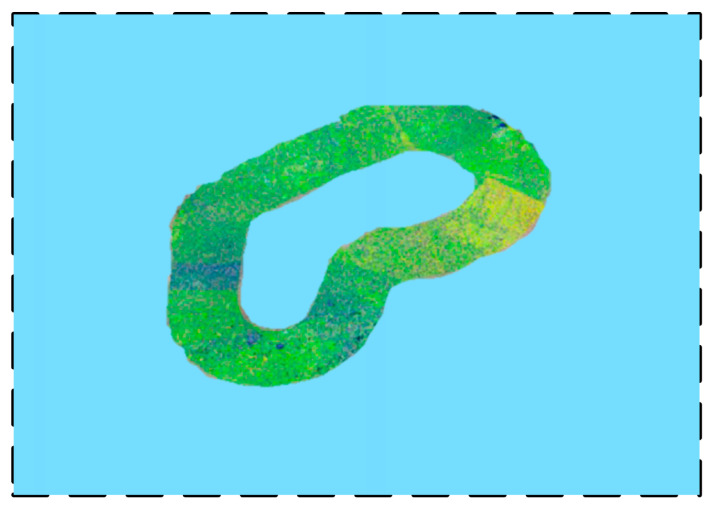
Robot’s traveling process corresponding to position information.

Repeating the above two steps, multiple sets of data can be obtained during the vehicle’s traveling. To better analyze and observe the movement of the vehicle, each position is shown in the 3D simulation field. As illustrated in Figure 12, the vehicle can be observed to have circumnavigated the GSSF.

As an illustrative example, Table 1 lists ten sets of pose information for mobile robots acquired from PTS. Each pose corresponds to a timestamp in the format X,Y,Z,w,φ, and κ.

To validate the accuracy of the 3D pose dynamic measurement system of the PTS, laser trackers are employed as ground truth for validation. That is, while the PTS tracks and measures the vehicle, the laser tracker also tracks the prism mounted on the vehicle, thereby measuring the pose of the vehicle (Figure 11). The results of the two are then compared. In Section 5, the pose data obtained by the PTS will be compared with the standard pose data obtained by the laser tracker.

#### 4.2.2. Static Validation

**Figure 13:**
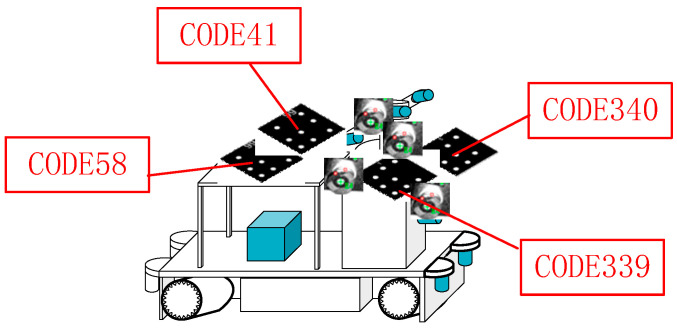
Scanning processing results of the image data obtained by PTS.

Given that there is only one laser tracker and that the technology of joint measurement by multiple laser trackers has not yet been sufficiently developed to be deployed in this context, the device is only capable of measuring multiple prisms on the vehicle in a sequential manner when the vehicle is stationary, thereby obtaining the pose of the vehicle. During the measurement process, the vehicle is required to move to a number of different positions within the GSSF. Once the vehicle has reached a stable state, the laser tracker is employed to ascertain its position. Subsequently, six high-precision PTS capture images, perform scanning processing, and calculate poses for the stationary mobile robot. Taking one PTS as an example, as shown in Figure 13, four coded targets were identified on the vehicle body, namely CODE58, CODE41, CODE340, and CODE339. 

Comparing the poses obtained from the two measurement devices, separately analyzing translation (x,y,z) and orientation angle (ω,φ,κ), the deviation for each pose can be obtained. This approach can be employed to indirectly assess and validate the accuracy of the pose measured statically by the PTS.

(7) $$E_{T}=\sqrt{(x-x_{0}{)}^{2}+(y-y_{0}{)}^{2}+(z-z_{0}{)}^{2}},$$

(8) $$\{\begin{array}{c} E_{\omega }=\omega -\omega_{0}\\ E_{\varphi }=\varphi -\varphi_{0}\\ E_{\kappa }=\kappa -\kappa_{0} \end{array},$$

In Equation (7), $E_{T}$ represents the deviation in translation, while x,y,z and $x_{0},y_{0},z_{0}$ are the translations obtained by the PTS and the laser tracker, respectively. In Equation (8), $E_{\omega }$, $E_{\varphi }$, and $E_{\kappa }$ represent the deviations in the three orientation angles, and ω, φ, and κ and $\omega_{0}$, $\varphi_{0}$, and $\kappa_{0}$ represent the orientation angles obtained by the PTS and the laser tracker, respectively.

#### 4.2.3. Dynamic Validation

**Figure 14:**
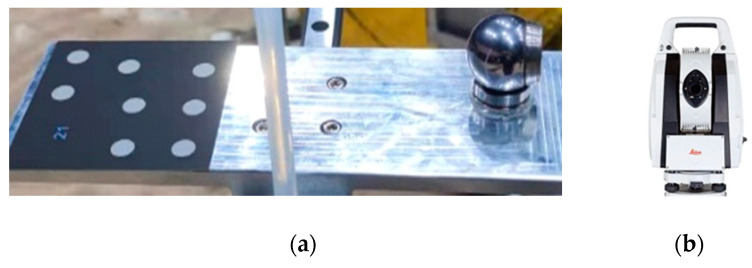
Tracking and comparison experiment of coded targets and prisms. (**a**) fixed prisms and coded targets. (**b**) laser tracker.

As illustrated in Figure 14, the experimental environment is situated within the simulation field. In the experimental site, a high-precision straight sliding rail, approximately 1.5 m in length, is fixed in place. A slider with a load structure is mounted on the sliding rail. When the sliding rail is inclined at a specific angle, the slider moves with the attached coded target and the prism of the laser tracker. 

A laser tracker is set up in the designated workspace. While the laser tracker measures the prism target sphere, it also measures the coded target, which allows it to obtain two sets of point data. The data obtained from the laser tracker and the PTS are separately fitted using the least squares method, thereby allowing the linear equation of the sliding rail to be obtained. The magnitude of the distance error from the point to the fitted line provides an indication of the actual position measurement deviation. It is expected that the theoretical trajectory of the measurement points obtained by the two measurement systems will remain collinear. Therefore, in this paper, the present study sought to assess the linear fitting accuracy of the motion trajectory and check its consistency, thereby indirectly evaluating and verifying the dynamic position measurement accuracy of the PTS.

The accuracy of fitting a straight line, designated as *s*, is quantified by the distance error between the points and the fitted line. If there are *m* points on the sliding rail that are involved in fitting the straight line *L*, and the perpendicular distance from the fitted points to *L* is $d_{i}$(i=1…m), then $s=\sqrt{\frac{1}{m}\sum_{i=1}^{m}{d_{i}}^{2}}$. In Equation (9), s is the linear fitting error calculated based on the data obtained by the laser tracker, $s^{\prime }$ is the linear fitting error calculated based on the data obtained by the PTS, and $E_{s}$ is their deviation.

(9) $$E_{s}=s-s^{\prime }$$

### 4.3. Accuracy Validation Method for Calibration of ROPs of Binocular Cameras Based on Epipolar Line Alignment

In stereo vision, the epipolar line serves a primary function in the purpose of fast searching and matching feature points, which can transform the two-dimensional (2D) search into one-dimensional (1D) search. In accurate epipolar images, corresponding points are distributed along the same horizontal line. The epipolar images are derived through epipolar correction based on the ROPs of the stereo camera or the absolute EOPs of each camera with respect to a certain spatial coordinate system, such as the vehicle reference frame. Consequently, epipolar images can be employed to assess the calibration accuracy of the intrinsic parameters and ROPs of the stereo camera. Prior to the generation of epipolar images, distortion correction is applied to the images in order to eliminate the impact of distortion on the generation of epipolar images.

The distortion calibration model presented in this paper is based on a ten-parameter calibration model [25]. The ten-parameter calibration model is effective in compensating for various predictable distortion errors and is currently the most widely used camera distortion model in digital industrial photogrammetry.

The complete ten-parameter intrinsic distortion model can be organized as in Equation (10):

(10) $$\left\{ \begin{array}{l} x^{\prime }=x-x_{0}+K_{1}\bar{x}r^{2}+K_{2}\bar{x}r^{4}+K_{3}\bar{x}r^{6}+P_{1}(r^{2}+2{\bar{x}}^{2})+2P_{2}\bar{x}\bar{y}+b_{1}\bar{x}+b_{2}\bar{y}\\ =\left(1+K_{1}r^{2}+K_{2}r^{4}+K_{3}r^{6}\right)\bar{x}+P_{1}({\bar{y}}^{2}+3{\bar{x}}^{2})+2P_{2}\bar{x}\bar{y}+b_{1}\bar{x}+b_{2}\bar{y}\\ y^{\prime }=y-y_{0}+K_{1}\bar{y}r^{2}+K_{2}\bar{y}r^{4}+K_{3}\bar{y}r^{6}+P_{2}(r^{2}+2{\bar{y}}^{2})+2P_{1}\bar{x}\bar{y}\\ \;=\left(1+K_{1}r^{2}+K_{2}r^{4}+K_{3}r^{6}\right)\bar{y}+P_{2}({\bar{x}}^{2}+3{\bar{y}}^{2})+2P_{1}\bar{x}\bar{y} \end{array}\right.,$$

where $\left(x^{\prime },y^{\prime }\right)$ represents the theoretical image plane coordinates, and (x,y) represents the actual imaging image plane coordinates, $\bar{x}=\left(x-x_{0}\right)\;$, $\bar{y}=\left(y-y_{0}\right)\;$, and $r^{2}={\bar{x}}^{2}+{\bar{y}}^{2}\;$. ($x_{0},y_{0}$) is the image principal point offset; $K_{1}$, $K_{2}$, and $K_{3}$ are the coefficients of radial distortion; $P_{1}$ and $P_{2}$ are the eccentricity distortion; and $b_{1}$ and $b_{2}$ are the coefficients of image plane distortion.

**Figure 15:**
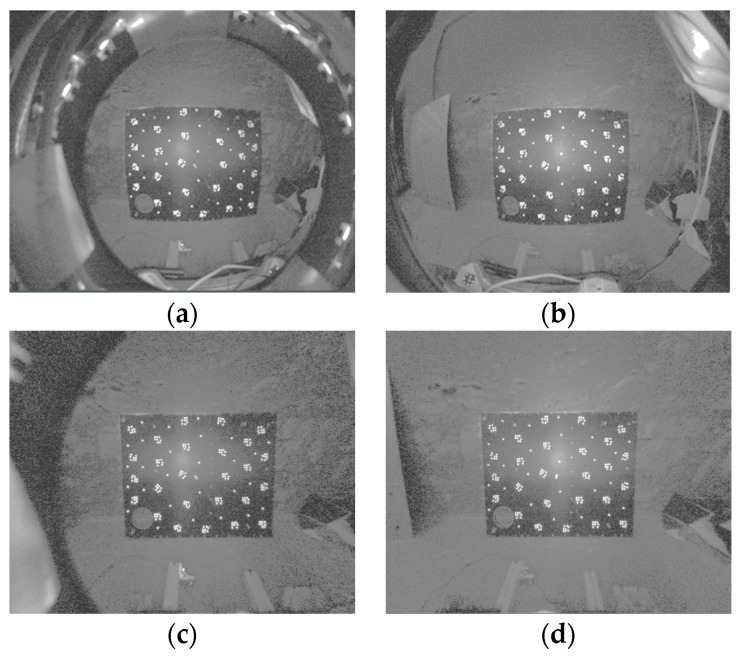
Distortion correction results of Hazcam images. (**a**,**b**) are the original left and right images of the Hazcam. (**c**,**d**) are the results after distortion correction.

As an illustrative example, the binocular Hazcam employs a ten-parameter calibration model to correct for distortion, resulting in undistorted images, as depicted in Figure 15.

**Figure 16:**
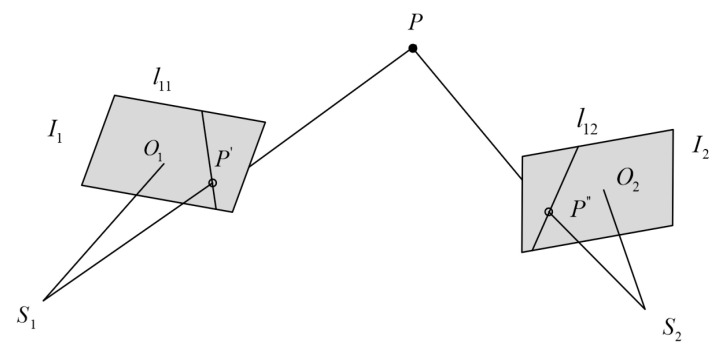
Epipolar geometry illustration.

Epipolar line rearrangement is a common method for generating epipolar images in aerial photogrammetry [26]. According to the geometric principles of epipolar lines, if the image coordinates of a specific object point are known in a given image, the corresponding image points on other images must be located on their respective epipolar lines, as illustrated in Figure 16. If the EOPs of each Navcam and Hazcam with respect to the vehicle coordinate system are known, the positions of these epipolar lines can be calculated.

Taking two camera stations as an illustrative example (Figure 16), $S_{1}$ and $S_{2}$ are the optical centers of each camera station, $I_{1}$ and $I_{2}$ are the respective image planes, $O_{1}$ and $O_{2}$ are the principal points of each image, $P^{\prime }$ and $P^{\prime \prime }$ are the corresponding image points of the object point P, and $l_{11}$ and $l_{12}$ are the corresponding epipolar lines for the image point $p^{\prime }$ on images $I_{1}$ and $I_{2}$.

Assume that the coordinate of a certain point in the image space coordinate system of station 1 is $\left(x_{1},y_{1},z_{1}\right)$ and the coordinate of this point in the image space coordinate system of station 2 is $\left(x_{2},y_{2},z_{2}\right)$, then the following formula (Formula (11)) is applicable.

(11) $$\left(\begin{array}{c} x_{2}\\ y_{2}\\ z_{2} \end{array}\right)=M_{2}^{T}\left(M_{1}\left(\begin{array}{c} x_{1}\\ y_{1}\\ z_{1} \end{array}\right)+\left(\begin{array}{c} X_{s1}-X_{s2}\\ Y_{s1}-Y_{s2}\\ Z_{s1}-Z_{s2} \end{array}\right)\right),$$

where $M_{1}$ and $M_{2}$ are the rotation matrices of camera stations 1 and 2 relative to the object space coordinate system, and $\left(x_{si},y_{si},z_{si}\right)$ is the translation parameters of camera stations 1 and 2 in the object space coordinate system (i = 1, 2). Moreover, since the coordinates of $S_{1}$ and $p^{\prime }$ are known in the image space coordinate system of camera station 1, denoted as (0,0,0) and $\left(x_{1},y_{1},-f\right)$, respectively. Therefore, according to Equation (11), it is possible to obtain the coordinates of $S_{1}$ and $p^{\prime }$ in the image space coordinate system of camera station 2, denoted as $\left(X_{s12},Y_{s12},Z_{s12}\right)$ and $\left(x_{s12},y_{s12},z_{s12}\right)$, respectively.

As the coplanarity of points $S_{1}$ and $p^{\prime }$ with any point on the epipolar line $l_{12}$, in the image space coordinate system of camera station 2, their plane equation can be represented as in Equation (12).

(12) $$\left|\begin{array}{ccc} x & y & z\\ X_{s12} & Y_{s12} & Z_{s12}\\ x_{12} & y_{12} & z_{12} \end{array}\right|=0,$$

In the image space coordinate system of camera 2, the plane equation for the image plane $I_{2}$ is

(13) z=−f,

Substitute Equation (13) into Equation (12) to obtain the equation for the epipolar line $l_{12}$ of the point $p^{\prime }$ on the image plane $I_{2}$.

(14) $$\left|\begin{array}{cc} Y_{s12} & Z_{s12}\\ y_{12} & z_{12} \end{array}\right|x-\left|\begin{array}{cc} X_{s12} & Z_{s12}\\ x_{12} & z_{12} \end{array}\right|y-\left|\begin{array}{cc} X_{s12} & Y_{s12}\\ x_{12} & y_{12} \end{array}\right|\cdot f=0,$$

Similarly, in the image space coordinate system of camera 2, the coordinates of $S_{2}$ are (0,0,0). According to Equation (14), the coordinates of $S_{2}$ in the image space coordinate system of camera 1 are $\left(X_{s21},Y_{s21},Z_{s21}\right)$, while in the image space coordinate system of camera 1, the coordinates of $p^{\prime }$ are $\left(x_{1},y_{1},-f\right)$. As the coplanarity of points $S_{2}$ and $p^{\prime }$ with any point on epipolar line $l_{11}$, in the image space coordinate system of camera 1, the corresponding plane equation can be expressed as Equation (15).

(15) $$\left|\begin{array}{ccc} x & y & -f\\ X_{s21} & Y_{s21} & Z_{s21}\\ x_{1} & y_{1} & -f \end{array}\right|=0,$$

Thus, the equation for the epipolar line $l_{11}$ of the point $p^{\prime }$ on the image plane $I_{1}$ can be expressed as

(16) $$\left|\begin{array}{cc} Y_{s21} & Z_{s21}\\ y_{1} & -f \end{array}\right|x-\left|\begin{array}{cc} X_{s21} & Z_{s21}\\ x_{1} & -f \end{array}\right|y-\left|\begin{array}{cc} X_{s21} & Y_{s21}\\ x_{1} & y_{1} \end{array}\right|\cdot f=0$$

**Figure 17:**
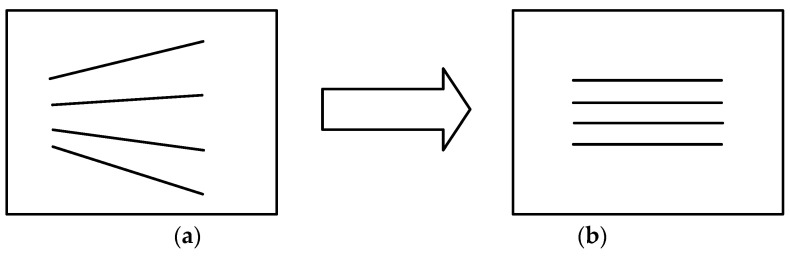
Comparison of images before and after epipolar line rearrangement. (**a**) Original image. (**b**) Epipolar image.

As shown in Figure 17, following the rearrangement of the epipolar lines, the coordinates of the left endpoint of the epipolar lines (Figure 17b) are identical to those of the left endpoint of the original epipolar lines (Figure 17a). By substituting the coordinates of the leftmost or rightmost column of the left image, Equations (14) and (16) can be employed to, respectively, obtain the equations of the original left and right epipolar lines. By applying a flat transformation to the original left and right epipolar lines pixel by pixel, the image pairs resulting from the rearrangement of the epipolar lines can be obtained.

### 4.4. Validation Method for Calibration Accuracy of EOPs and Structural Robustness for Individual Sensors Based on Point Cloud Projection and Superposition

#### 4.4.1. Validation of Camera-Based Sensors Based on Point Cloud Projection

For camera-based sensors such as Navcams, Hazcams, and multispectral cameras, the point cloud obtained from the GSSF can be re-projected onto the camera plane to verify the accuracy of the camera’s calibrated EOPs and structural robustness [27]. The projection follows the principle of collinearity equations, whereby the laser scanning 3D points are considered to be object coordinates. The 3D points are projected onto the camera plane using the camera’s EOPs in the object coordinate system of GSSF. In Equation (3), $\left(X_{A},Y_{A},Z_{A}\right)$ represents the coordinates of the laser’s 3D points of GSSF, f is the camera’s focal length, and $X_{S}$, $Y_{S}$, $Z_{S}$, ω, φ, and κ are the camera’s EOPs in the laser point cloud’s object coordinate system.

The projection of the 3D point cloud onto the image plane involves two scenarios. The first scenario is to re-project the point cloud onto the original image, where distortion correction is applied to the projected points prior to their projection onto the image plane. The second scenario is to project the point cloud directly onto an undistorted image, thereby eliminating the need for distortion correction for the projected points. This paper employs accuracy validation through projection onto the original images. The specific validation is divided into two distinct categories: qualitative and quantitative methods. For qualitative validation, the benchmark point cloud is re-projected onto the camera plane and compared with the prominent edges in the image. Typically, local edges of objects can be magnified, and the accuracy of the projection can be observed visually at the pixel level. For quantitative validation, distinctive corresponding points are manually selected on both the 3D point cloud and the image, respectively, and the error between the ideal re-projected 2D points and the actual 2D points is calculated.

In this paper, the benchmark used for validation is the laser scanning point cloud in the true north coordinate system. As the point cloud is acquired in all directions without blind spots, rather than under the current camera perspective, it must undergo automatic filtering to exclude points that do not participate in the current camera’s field of view.

**Figure 18:**
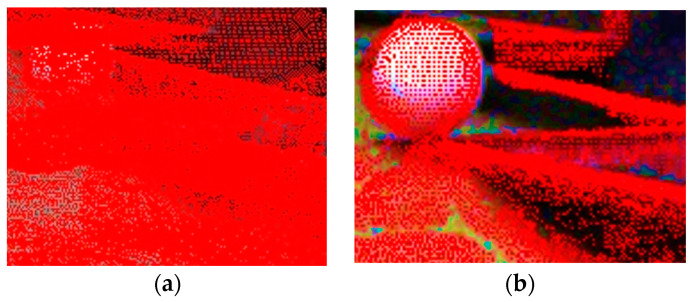
Comparison of the effect of with and without excluding non-visible points in projection. (**a**) Projection without excluding non-visible points; (**b**) projection while excluding non-visible points.

Invalid points that require elimination are divided into two categories. The first category comprises points that are beyond the current camera’s field of view when the mobile robot is moving forward. The second category comprises the points that are obscured by the object and, therefore, not directly visible. The points of GSSF are stitched together from multiple angles to form a complete point cloud. However, when the current camera is re-projected, it can only visualize the front side of a 3D object. For example, the camera can only see the front of the rock but not the back. These points on the back belong to non-visible points. Consequently, if these non-visible points are not excluded, they will be re-projected onto the camera plane, resulting in the superposition of multiple points on the forward-visible points. This could potentially impact the visual effect of the projection, leading to misinterpretations. As illustrated in Figure 18, this can result in visual misjudgments. Consequently, it is imperative to exclude non-visible points.

The automatic removal of non-visible points necessitates the preliminary construction of a fine triangular mesh model of the simulated terrain. The visibility discrimination is then performed by determining whether the line of sight between the current laser point and the camera passes through the triangular network or not.

(a) Construction of a fine mesh for GSSF based on laser scanning point cloud

The greedy triangulation projection is an algorithm that can perform rapid triangulation reconstruction from the point cloud [28]. This algorithm is most effective in scenarios where the model’s surface is relatively smooth and the point cloud density varies in a relatively uniform manner. The fundamental concept underlying the greedy triangulation projection is as follows. Firstly, compute the normals of the point cloud data and then project the 3D point cloud onto a 2D plane through the normals. Subsequently, triangulation is performed in the 2D plane according to the positions of the projected points using the Delaunay-based spatial region-growing algorithm. The triangular mesh in 3D space is derived from the obtained topological relationships between points and the outcome of triangulation in the 2D plane. This process ultimately yields the reconstructed 3D model.

(b) Automatic removal of non-visible 3D points for the camera

**Figure 19:**
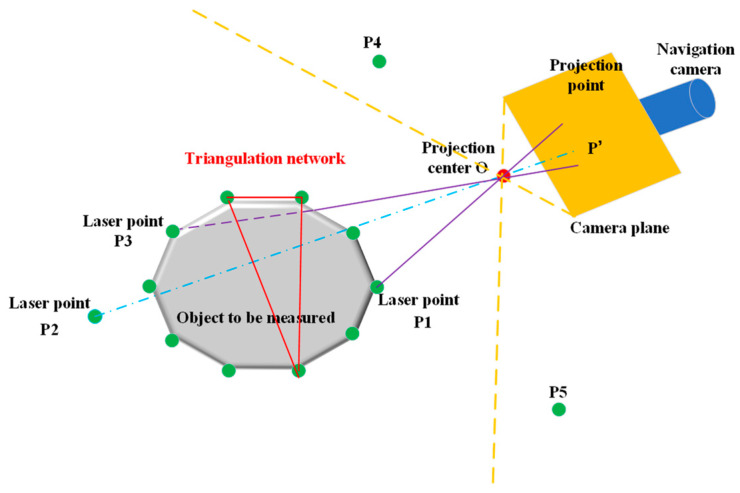
The removal of points that are out of field of view and non-visible.

As illustrated in Figure 19, *P*_1_, *P*_2_, *P*_3_, *P*_4_, and *P*_5_ are discrete points in the 3D laser scanning point cloud. These points are situated on the surface of, or behind, the object to be measured or outside the camera’s field of view. For the Navcam, points *P*_4_ and *P*_5_ are outside the field of view and, therefore, cannot be imaged onto the image plane. Consequently, they must be removed. *P*_1_ is directly visible to the camera as it is in the forward direction, while *P*_2_ is behind the object and is not directly visible. However, its projection on the image plane can affect visual interpretation and, therefore, requires removal. Although *P*_3_ is situated on the object’s surface, it is occluded by the triangular mesh formed by the front points. Consequently, *P*_3_ is also considered a non-visible point and must be removed automatically.

**Figure 20:**
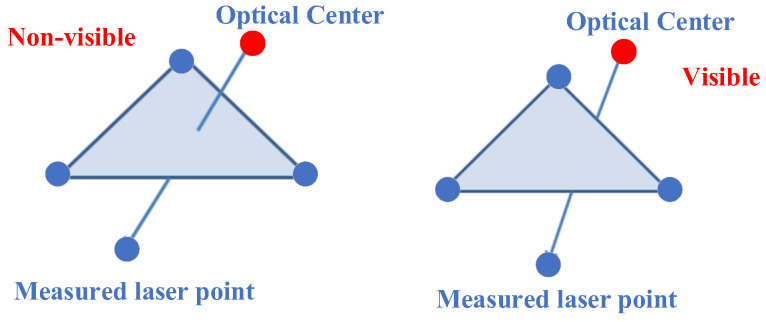
Ray–triangle intersection for fast visibility judgment.

(1) The removal of *P*_4_ and *P*_5_. *P*_4_ and *P*_5_ are projected onto the image plane. The coordinates (X,Y,Z) are transformed into 2D image plane coordinates (x,y) by means of the collinearity equation. The 2D coordinates (x,y) are subsequently transformed into pixel coordinates (U,V). Judging the relationship between U, V, and the width and height of the image, if U > width or V > height, the two points lie outside the field of view and can be eliminated.

(2) The removal of *P*_2_ and *P*_3_. It is necessary to construct a triangular mesh for all discrete points. The construction of triangular faces enables the computation of intersections between the lines connecting *P*_2_ or *P*_3_ to the camera center, and the faces of the mesh are computed. Subsequently, the removal is then carried out based on visibility checks, as illustrated in Figure 20.

In this study, the calculation of visibility utilizes a method referring to the proposal of Yi M et al. [29]. In their research, they employ a fast, minimal-storage ray–triangle intersection detection algorithm to determine the intersectionality of vectorial lines with triangles in 3D space, thus realizing the visibility judgment of two points with known triangles in 3D space. The center of the camera is regarded as a point in space, and the laser point on the ground is similarly regarded as another point. The line connecting the camera center and the measured laser point is considered a ray in space, and the surrounding laser points construct spatial triangles. By iterating through all triangles in the triangulation network, the intersection of rays with space triangles is judged one by one, and then the visibility is obtained. Non-visible laser points can be automatically removed, thereby providing a clean data source for the subsequent point cloud projection.

#### 4.4.2. Validation of Non-Camera-Based Sensor Using Point Cloud Superposition

For sensors that are not camera-based, such as TOF, the calibration of intrinsic and extrinsic parameters is achieved through the capture of photos during the calibration process. However, during the actual operation, no photos were taken; only point cloud data were obtained. In contrast, LiDAR only acquires the point cloud throughout the process. For TOF and LiDAR, point cloud projection can also be employed to validate the intrinsic and extrinsic parameters by re-projecting them to the left Navcam and comparing them with the image edges. However, since the point cloud acquired by TOF is sparser, the effect is less pronounced when the point cloud acquired by TOF is re-projected to an image. Moreover, due to the compact nature of LiDAR, the acquired point cloud is notably sparser, rendering the projection onto an image highly ineffective. Concurrently, this methodology is susceptible to interference from the Navcam and can only verify the relative relationship between the Navcam and TOF or LiDAR, rendering it incapable of validating the absolute EOPs and structural robustness of TOF and LiDAR with respect to the vehicle.

Consequently, both the GSSF point cloud and the TOF or LiDAR point cloud can be transferred to the vehicle body coordinate system to ascertain their superimposed coincidence. This process can also serve to verify the external parameter calibration accuracy and structural robustness of TOF or LiDAR from the side.

## 5. Experiments and Analysis

### 5.1. Accuracy Evaluation of Constructed 3D GSSF

A laser tracker is employed as a standard reference device for the measurement of vehicle body position. The laser tracking system selected is the Leica AT403, Hexagon Corporation, Nasdaq Stockholm, Sweden [30], which has a maximum angular measurement accuracy of 0.5″. The static absolute coordinate measuring accuracy is 7 μm + 3 μm/m, while the dynamic absolute coordinate measuring accuracy is 15 μm + 6 μm/m. During on-site testing, the tracking system is typically positioned at a distance of no more than 6 m from the target. Therefore, the maximum system error of the tracking system can be considered to be 7 μm + 3 μm/m × 6 m = 25 μm, which can be utilized as the true value for testing purposes.

A 3D laser scanner is employed as an acquisition device for ground reference point cloud data. The ground 3D laser scanner selected is the Z + F Imager 5016, Zoller + Fröhlich Corporation, Wangen im Allgäu, Germany [31]. The scanner’s accuracy within a range of 10 m can reach 0.3 mm. The spatial resolution of acquired points is, on average, 0.005 m. The device is equipped with a white spherical target, which allows for the spherical fitting of the point cloud in order to obtain the sphere’s center. This enables the manual stitching of multi-station point clouds. Based on the experimental conditions and the generation principle of the 3D GSSF outlined in Section 4.1, a simulation field for the ground surface is constructed.

#### 5.1.1. Absolute Accuracy of Terrain 3D Points

**Figure 21:**
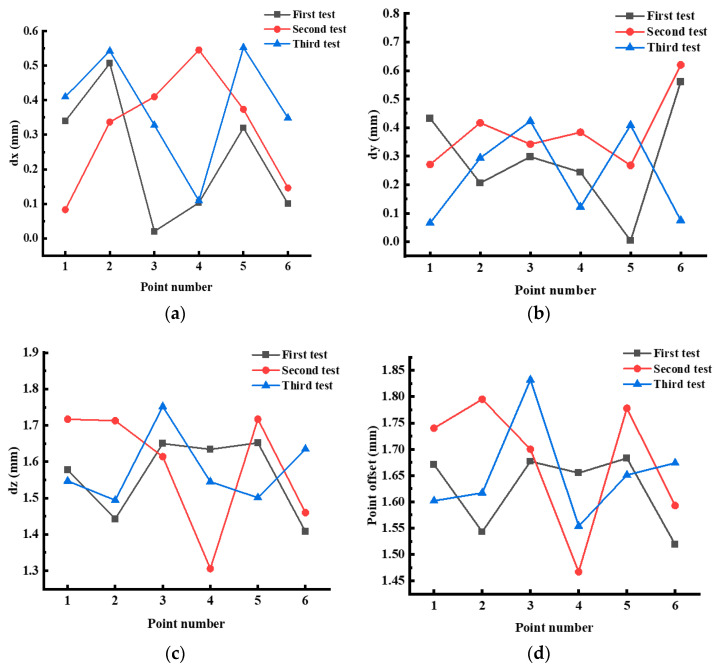
Absolute accuracy of terrain 3D points. (**a**) Deviation in the X direction. (**b**) Deviation in the Y direction. (**c**) Deviation in the Z direction. (**d**) Point deviation.

The absolute accuracy of the terrain 3D points can be calculated using Equation (1), which is shown in Figure 21, after three experiments in which six spherical targets are used as six 3D points to be tested. Due to the discrepancies in the positioning of the five stations established during the scan operation on three occasions, the angle of the three scans and the quality of the point cloud are also somewhat disparate. Consequently, the accuracy of the laser point cloud when fitted to several spherical targets is also disparate, resulting in discrepancies in the test errors of the six standard points of the three tests.

Nevertheless, the maximum deviations for the three tests were 0.507 mm, 0.546 mm, and 0.553 mm in the X-direction; 0.561 mm, 0.620 mm, and 0.423 mm in the Y-direction; 1.652 mm, 1.717 mm, and 1.752 mm in the Z-direction; and 1.683 mm, 1.795 mm, and 1.832 mm in the 3D point locations, respectively. It can be observed that the maximum deviation remains relatively stable in both the three spatial sub-directions and the point positions. This indicates a high degree of accuracy in the reference points, which indirectly reflects the high accuracy of the absolute 3D laser point used for terrain construction.

#### 5.1.2. Accuracy of the Reconstructed Model

The GSSF is equipped with twelve standard scale bars, with five having a calibrated length of 600 mm (Nos. 1 to No. 5) and the remaining seven scale bars having a length of 1100 mm (Nos. 6 to No. 12) (as illustrated in Figure 9). The GSSF is reconstructed, and in the reconstructed 3D scene, the measured length of the standard scale bars is compared with their calibrated length to evaluate the reconstruction model’s accuracy. If the discrepancy is less than 1cm, the accuracy of terrain reconstruction is better than 1 cm.

Standard scale bars 803-MCP, Brunson Instrument Company, Kansas City, MO, USA [32] were used in the test, with an accuracy better than 5 μm after calibration.

**Figure 22:**
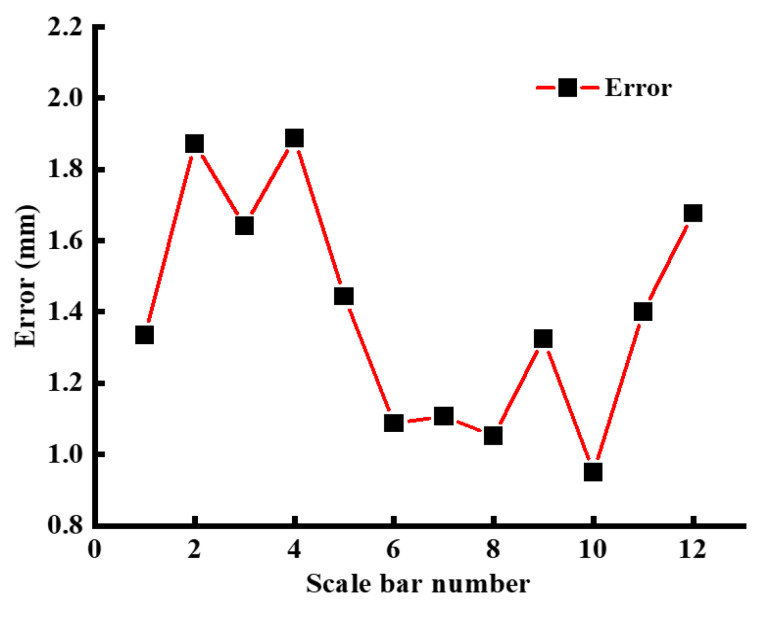
Static terrain reconstruction accuracy.

By means of Equation (2), the accuracy of the reconstructed terrain can be calculated. Figure 22 illustrates that the maximum static error in terrain is 1.887 mm, which is indicative of a high degree of accuracy in 3D reconstruction.

The results of the accuracy tests conducted on the terrain 3D points and 3D models described above demonstrate that the terrain simulated by the high-precision 3D laser scanner in this study exhibits high accuracy and can serve as a high-precision reference for validation.

### 5.2. Accuracy Assessment of the Pose Measurement of PTS

#### 5.2.1. Accuracy Evaluation under Static Verification

The pose data obtained by the laser tracker are regarded as the truth value. The poses obtained from the PTS and the laser tracker were compared, and fifteen sets of data were selected for statistical analysis. As an illustrative example, Table 2 presents the measurement deviations for each pose, in which the translation values (X,Y,Z) and orientation angles (ω,φ,κ) were analyzed separately.

It can be observed that the maximum translation deviation is 0.789 mm, and the maximum orientation angle deviation is 0.0987°, indicating that the tracking measurement accuracy of the PTS is sufficiently high.

#### 5.2.2. Accuracy Evaluation under Dynamic Verification

**Figure 23:**
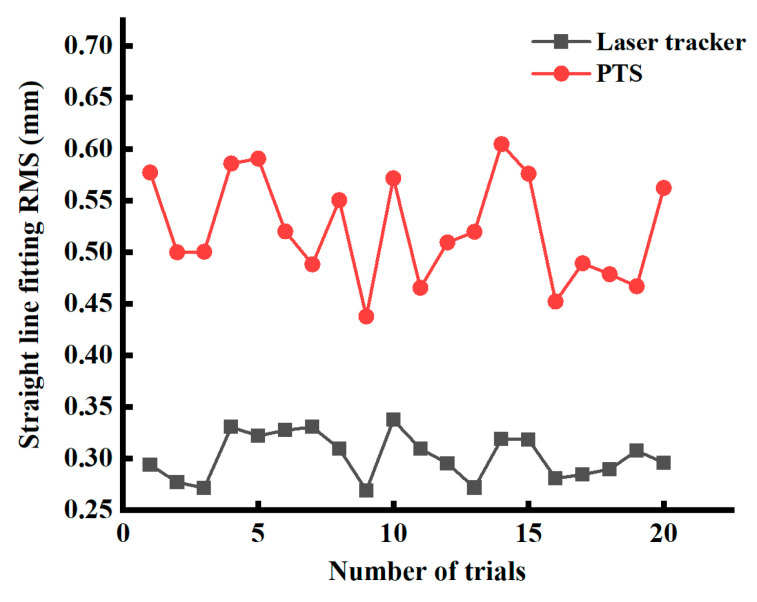
Comparisons of dynamic trajectory linearity error.

Twenty sets of linear fitting errors were obtained by conducting twenty sliding tests on the sliding rail separately with the PTS and laser tracker. Figure 23 depicts the twenty sets of linear fitting errors.

Figure 23 illustrates the maximum linear fitting errors of the laser tracker and the PTS for the sliding rail. The respective values are 0.337 mm and 0.605 mm, with a maximum deviation of 0.286 mm between the linear fitting errors of the two systems. Taking into account the systematic error of the measurement system of the laser tracker and the vectorial nature of the 3D positioning error, the dynamic measurement error of the system of the PTS will not exceed 0.605 mm + 0.045 mm = 0.650 mm. It can be observed that compared with the laser tracker, the linear fitting deviation of the measurement system of the PTS is relatively stable within 0.3 mm, which is relatively high in measurement accuracy and robustness.

### 5.3. Evaluation of the Calibration Accuracy of ROPs for Binocular Navcams and Hazcams

**Figure 24:**
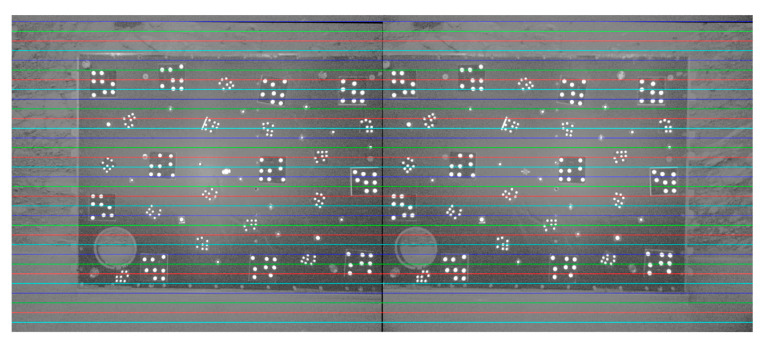
Epipolar image pair of Navcam.

**Figure 25:**
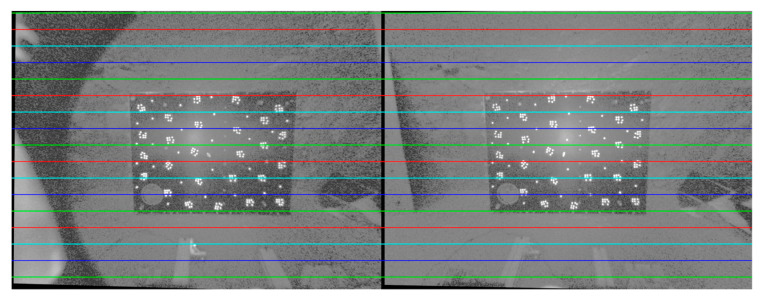
Epipolar image pair of Hazcam.

The epipolar line realignment algorithm enables the correction of epipolar line distortion based on two sets of navigation binocular camera and hazard avoidance binocular camera data, which have been processed to remove distortion. This results in the generation of epipolar image pairs. The internal and external parametric accuracy of the binocular camera is verified by observing the alignment of the epipolar line, as illustrated in Figure 24 and Figure 25.

As illustrated by the above two groups of data presented, the overall alignment of the epipolar line is satisfactory, reflecting the accuracy of the internal and external parametric calibration results.

Subsequently, the two groups of images following epipolar correction were utilized as the research subjects, with the Speeded Up Robust Features (SURF) [33] algorithm employed for feature point matching. To enhance the accuracy of the algorithm, the Random Sample Consensus (RANSAC) [34] was also employed to further refine the initial matched point pairs, thereby yielding more accurate statistical data results. Table 3 presents the error in Y coordinates of matching points for each epipolar image pair in the two datasets.

It can also be said that Table 3 illustrates the degree of alignment accuracy achieved through the matching of feature points. Given that the adopted images have undergone epipolar correction, it can be assumed that the matched point pairs will lie on the same horizontal line (i.e., with equal *y* values). Consequently, the alignment accuracy of the epipolar line is calculated based on the differences in the *y* coordinates of the matched point pairs. The average error is $\frac{1}{n}\sum \left|y_{1}-y_{2}\right|$, the RMSE is $\sqrt{\frac{1}{n}{\sum \left(y_{1}-y_{2}\right)}^{2}}$, and the maximum error is $\max\{\left|y_{1}-y_{2}\right|\}$, where *n* represents the number of matched point pairs.

The results presented in Table 3 demonstrate that the average error is less than 0.9 pixels, the maximum error is less than 2 pixels, and the RMSE is approximately 1 pixel. These findings illustrate the high degree of accuracy associated with the relative orientation of the Navcam/Hazcam and the accuracy of epipolar images.

### 5.4. Evaluation of Calibration Accuracy of EOPs and Structural Robustness for Individual Sensors

This section is responsible for the validation of the calibration accuracy and structural robustness of the EOPs, which include the Navcam, Hazcam, multispectral camera, TOF, and LiDAR.

#### 5.4.1. Validation of EOPs’ Calibration Accuracy and Structural Robustness for Camera-Based Sensors

A selection of data from a group of left Navcams, a group of left Hazcams, and a group of multispectral cameras is chosen for testing and validation, respectively. In this test, the laser reference point cloud coordinates are first transformed into the current camera coordinate system using the external parameters of the camera coordinate system in the true north coordinate system. During the preprocessing stage, points that fall outside the camera’s field of view are automatically removed, thus ensuring that the resulting point clouds are confined to the field of view, reducing unnecessary computational load.

**Figure 26:**
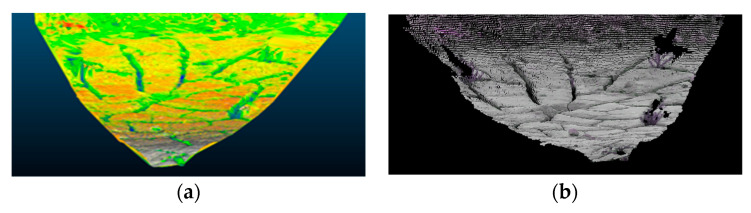
Terrain mesh and the visible point cloud. (**a**) Terrain mesh. (**b**) Visible point cloud.

Subsequently, a greedy triangulation projection mesh method is employed to generate a mesh of the point clouds within the field of view, as illustrated in Figure 26a. The triangulation mesh generated by the greedy triangulation projection algorithm conforms to the actual terrain and serves as the basis for subsequent visibility determination. A point-by-point visibility judgment is made with the camera, and non-visible laser points are automatically rejected to obtain a visibility point cloud, as shown in Figure 26b.

**Figure 27:**
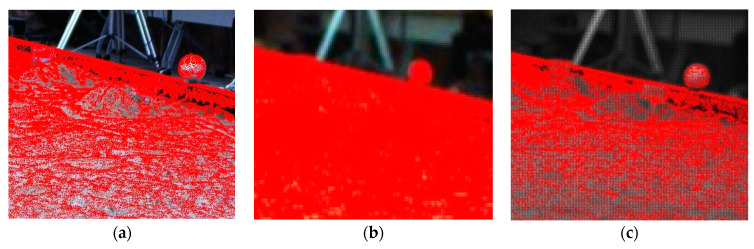
Qualitative evaluation of the point cloud projection. (**a**) Left Navcam; (**b**) left Hazcam; (**c**) multispectral camera.

It can be observed that the removal of non-visible points results in the deletion of laser points behind terrain objects, such as rocks and hills. This process ensures that these points do not interfere with the projection onto the camera plane, thereby guaranteeing clear object edges, as illustrated in Figure 27.

Upon meticulous examination of the projection results, it can be observed that the outline of the wooden board on the slope and the white spherical targets at the edge of the simulation field are distinctly discernible. Moreover, the conformity of the projected bodies to the actual bodies is quite good, with an accuracy of approximately 1–2 pixels.

Furthermore, a quantitative evaluation of the projection results was conducted through the manual selection of four corresponding points on each group of images and point clouds. Following the completion of the quantitative calculations, the projection error for corresponding points was found to be approximately 2 pixels (refer to Table 4). Qualitative and quantitative assessments indicate that the calibration accuracy of this system is relatively high and that the structure of the multi-sensor system is comparatively stable.

#### 5.4.2. Validation of EOPs’ Calibration Accuracy and Structural Robustness for Non-Camera-Based Sensors

**Figure 28:**
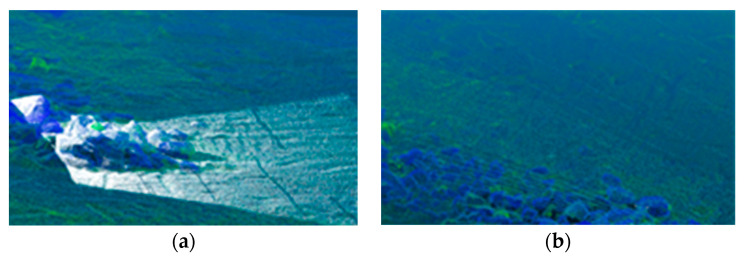
Superposition of non-camera-based sensor point clouds with laser-scanning point clouds. (**a**) TOF overlaid with laser scanner. (**b**) LiDAR overlaid with laser scanner.

In Figure 28, the reference point cloud is acquired by the laser scanner. In Figure 28a, the white point cloud represents data acquired by the TOF, while in Figure 28b, the LiDAR point cloud appears as white lines. It can be observed that the TOF point cloud is denser than the LiDAR point cloud, indicating a greater degree of conformity with the reference point cloud. The LiDAR point cloud is sparser in comparison to the reference point cloud, yet it visually aligns well with the latter.

As the aforementioned tests are rather trivial, we provide a concise summary in a single paragraph. In the evaluation of the GSSF’s accuracy, the spatial accuracy of the terrain 3D points is found to be better than 1.832 mm, while the accuracy of the static terrain reconstruction is better than 1.887 mm. This indicates that the 3D simulation field is constructed with high accuracy. In the evaluation of the pose measurement accuracy of the high-precision PTS, the translation accuracy obtained through static verification was found to be better than 0.789 mm, while the pose angle accuracy was better than 0.0987°. Furthermore, the point measurement accuracy obtained through dynamic verification was found to be better than 0.75 mm. This evidence demonstrates that the tracking and measurement accuracy of the PTS is sufficiently high and that the system is stable. In the evaluation of calibration accuracy of binocular ROPs, the accuracy is approximately one pixel on the epipolar line alignment, indicating that the relative relationship between the binocular Navcams and Hazcams is accurate and reliable. In the evaluation of the EOPs calibration accuracy and structural robustness of the individual sensors, the qualitative and quantitative results based on the projection of the point cloud demonstrate that the accuracy is approximately 2 pixels. Furthermore, the point cloud of TOF and the point cloud of the LiDAR exhibit a high degree of coincidence with the baseline point cloud of GSSF, which indicates a high accuracy of the EOPs of the various types of sensors calibrated and a high degree of structural robustness.

In order to provide a more comprehensive overview of the evaluation precision categories involved in the experiment and their respective roles, we have added a summary table (Table 5).

## 6. Conclusions

This study presents an innovative ground–object–air cooperation mechanism, designated as “GSSF-Robot-PTS”, which is unified within a true north datum coordinate system. This mechanism interconnects the static GSSF, the PTS responsible for real-time tracking and measurement in the air, and the real-time traveling mobile robot on the ground. A set of dynamic validation systems for calibration accuracy and structural robustness of multi-sensor mobile robots is proposed, which addresses the existing literature gap on the validation method for calibration accuracy and structural robustness of the whole vehicle sensor system of mobile robots. Furthermore, the proposed system will serve as a reliable reference for the calibration accuracy and structural robustness of sensors in indoor tests and validations for various mobile robots.

## Figures and Tables

**Table 1:** Partial display of pose data of the mobile robot acquired from PTS.

	Timestamp	*X* (m)	*Y* (m)	*Z* (m)	w (°)	φ (°)	κ (°)
1	26,590,031	−9.5154	13.8381	0.9961	0.2582	359.7366	342.5750
2	26,590,098	−9.5157	13.8382	0.9959	0.2483	359.7115	342.5742
3	26,590,164	−9.5158	13.8382	0.9959	0.2466	359.7072	342.5741
4	26,590,231	−9.5155	13.838	0.9961	0.2606	359.7285	342.5785
5	26,590,298	−9.5154	13.8381	0.9961	0.2561	359.7469	342.5753
6	26,590,364	−9.5154	13.838	0.9961	0.2597	359.7413	342.5760
7	26,590,431	−9.5154	13.838	0.9961	0.2621	359.7377	342.5764
8	26,590,498	−9.5157	13.8381	0.9959	0.2564	359.7141	342.5755
9	26,590,564	−9.5158	13.8381	0.9959	0.2529	359.6992	342.5750
10	26,590,631	−9.5154	13.838	0.996	0.2641	359.7475	342.5779

**Table 2:** The accuracy of pose measurement of PTS.

Serial No.	Deviation of Translation (mm)				Deviation of Orientation (°)		
	ΔX	ΔY	ΔZ	Total Deviation	Δω	Δφ	Δκ
1	0.1269	0.2000	0.1733	0.2935	0.0592	0.0589	0.0832
2	0.1747	0.1937	0.2342	0.3506	0.0929	0.0695	0.0689
3	0.2584	0.2484	0.2854	0.4582	0.0970	0.0813	0.0662
4	0.4413	0.4424	0.4818	0.7890	0.0696	0.0694	0.0585
5	0.2855	0.1950	0.2753	0.4419	0.0582	0.0832	0.0978
6	0.3339	0.3324	0.3675	0.5975	0.0820	0.0539	0.0737
7	0.1467	0.1707	0.1906	0.2949	0.0940	0.0913	0.0581
8	0.2682	0.2759	0.2931	0.4837	0.0919	0.0574	0.0706
9	0.2543	0.2318	0.2533	0.4272	0.0608	0.0772	0.0867
10	0.2596	0.2792	0.2919	0.4802	0.0745	0.0863	0.0744
11	0.3390	0.3669	0.3522	0.6112	0.0632	0.0542	0.0693
12	0.3235	0.2835	0.3118	0.5313	0.0808	0.0987	0.0829
13	0.4342	0.3964	0.3893	0.7052	0.0890	0.0981	0.0979
14	0.3448	0.3299	0.3427	0.5875	0.0680	0.0585	0.0550
15	0.2586	0.1838	0.1994	0.3747	0.0691	0.0863	0.0890

**Table 3:** Error in Y coordinates of matching points for each epipolar image pair (unit: Pixels).

Epipolar Image Pair	Number of Matching Points	Mean Error	RMSE	Maximum Error
Navcam	4994	0.727	0.769	1.997
Hazcam	356	0.838	0.996	1.980

**Table 4:** Quantitative evaluation of corresponding point projection.

	Navcam (px)	Hazcam (px)	Multispectral Camera (px)
RMSE	2.42	2.12	1.66

**Table 5:** Summary of different categories of evaluation precision.

Categories of Precision Evaluation	Roles	Evaluation Methods	Accuracy
GSSF	Providing a baseline 3D terrain	Terrain 3D points	<1.832 mm
		Reconstructed surface	<1.887 mm
PTS	Pose measurement of the robot	Static verification	Translation: <0.789 mm; Pose angle: <0.0987°;
		Dynamic verification	Linear fitting: <0.75 mm
Calibrated ROPs for Binocular Cameras	Validating the relative relationship between the binocular Navcams/Hazcams	Epipolar line alignment	<1 pixel
Calibrated EOPs and Structural Robustness for Individual Sensors	Validating the relative relationship between each sensor relative to the vehicle body	Projection of the point cloud for camera-based sensors	<2 pixels
		Superposition of the point cloud for non-camera-based sensors	Looks good visually

## Data Availability

The original contributions presented in the study are included in the article, further inquiries can be directed to the corresponding author.
